# Ferroptosis in head and neck squamous cell carcinoma: from pathogenesis to treatment

**DOI:** 10.3389/fphar.2024.1283465

**Published:** 2024-01-19

**Authors:** Jing Yang, Zhaowei Gu

**Affiliations:** Department of Otolaryngology Head and Neck Surgery, Shengjing Hospital of China Medical University, Shenyang, China

**Keywords:** head and neck squamous cell carcinoma, ferroptosis, pathogenesis, GSH, GXP4, treatment

## Abstract

Head and neck squamous cell carcinoma (HNSCC) is the sixth most common malignant tumor worldwide, with high morbidity and mortality. Surgery and postoperative chemoradiotherapy have largely reduced the recurrence and fatality rates for most HNSCCs. Nonetheless, these therapeutic approaches result in poor prognoses owing to severe adverse reactions and the development of drug resistance. Ferroptosis is a kind of programmed cell death which is non-apoptotic. Ferroptosis of tumor cells can inhibit tumor development. Ferroptosis involves various biomolecules and signaling pathways, whose expressions can be adjusted to modulate the sensitivity of cells to ferroptosis. As a tool in the fight against cancer, the activation of ferroptosis is a treatment that has received much attention in recent years. Therefore, understanding the molecular mechanism of ferroptosis in HNSCC is an essential strategy with therapeutic potential. The most important thing to treat HNSCC is to choose the appropriate treatment method. In this review, we discuss the molecular and defense mechanisms of ferroptosis, analyze the role and mechanism of ferroptosis in the inhibition and immunity against HNSCC, and explore the therapeutic strategy for inducing ferroptosis in HNSCC including drug therapy, radiation therapy, immunotherapy, nanotherapy and comprehensive treatment. We find ferroptosis provides a new target for HNSCC treatment.

## 1 Introduction

Head and neck squamous cell carcinoma (HNSCC) is the sixth most common malignant tumor worldwide, with high morbidity and mortality. According to “Estimating the global cancer incidence Current Oncology Reports,” 890,000 new cases and 450,000 deaths from HNSCC were reported worldwide in 2018 (Bray et al., 2018). Morbidity and mortality rates are increasing every year. In 2020, 930,000 new cases and 470,000 deaths due to HNSCC were reported worldwide (Sung et al., 2021). The majority of HNSCCs occur in the mucosa of the oral cavity, nasopharynx, oropharynx, hypopharynx, and larynx. Tobacco, alcohol, betel nuts, and viral infections are the main risk factors for HNSCC. The prevalence of HNSCC has grown slowly in recent years owing to anti-smoking policies, increased awareness of risk factors, and improved self-care (Dayyani et al., 2010). However, the incidence of HNSCC (mainly oropharyngeal cancer) has gradually increased owing to human papillomavirus (HPV) infections (Gillison et al., 2000; Gillison, 2004; Ragin et al., 2007). Oropharyngeal cancer is usually associated with HPV infection, and its prognosis may depend on whether the patient with HNSCC also has HPV or not (Isayeva et al., 2012; Michaud et al., 2014; Stein et al., 2015). Well-known oncogenes in high-risk subtypes of HPV include the E6 and E7 oncogenes. HPV E6 and E7 DNA testing has been used to screen for HPV^+^ tumor growth and recurrence and is an indicator of early treatment and testing programs (Chuang et al., 2008; Fakhry et al., 2019). Epstein-Barr virus (EBV) infection is often associated with nasopharyngeal cancer. Chan et al. found that the sensitivity and specificity of plasma EBV DNA detection for nasopharyngeal carcinoma diagnosis were 97.1% and 98.6%, respectively (Chan et al., 2017). At present, certain pro-apoptotic drugs are used in HNSCC, although they often lead to drug resistance (Carneiro and El-Deiry, 2020). This warrants the study of other mechanisms of cell death to combat tumor development. Ferroptosis is an important mode of cell death that can be activated by various types of cancer or combination therapies, including chemotherapy, radiotherapy, immunotherapy, and targeted therapy (Sun et al., 2016a; Guo et al., 2018; Lei et al., 2020). Therefore, an in-depth understanding of the molecular mechanism of ferroptosis in HNSCC may provide a new target for the treatment of HNSCC.

Dolma (Dolma et al., 2003) discovered a new compound in 2003 called erastin, which induces cell death. Unlike other forms of cell death, during erastin-mediated cell death, the nuclear morphology does not change and cannot be reversed by caspase inhibitors. This indicates that erastin induces a new form of cell death. This type of cell death elicits different morphological characteristics compared to apoptosis, such as chromatin condensation, cell shrinkage, formation of apoptotic bodies, and formation of autophagosomes (Agmon et al., 2018; DeHart et al., 2018; Li et al., 2020), which are key features of autophagy (Zhou et al., 2019). The most distinct morphological features are mitochondrial atrophy, reduced mitochondrial size with increased membrane density, rupture of the mitochondrial outer membrane, and a reduced number of mitochondrial cristae (Stockwell et al., 2017). This cell death process was officially named ferroptosis in 2012 (Dixon et al., 2012).

Ferroptosis is typically characterized by the excessive accumulation of iron and the subsequent production of large amounts of reactive oxygen species (ROS). Ferroptosis can be inhibited by antioxidants and iron chelators but not by inhibitors of autophagy, pyroptosis, and apoptosis. Ferroptosis is associated with the occurrence and development of various diseases, such as tumor infection, immune diseases, neurodegeneration, and tissue damage. Research on the occurrence, development, and treatment of cancer has recently become increasingly extensive. In particular, ferroptosis inducers can effectively kill tumors (Huang et al., 2021). This article will illustrate the molecular mechanism of ferroptosis and the progress of HNSCC treatment, including chemotherapy, radiotherapy, and tumor immunotherapy, as well as provide new strategies for the treatment of HNSCC.

## 2 Mechanism of ferroptosis

**FIGURE 1:**
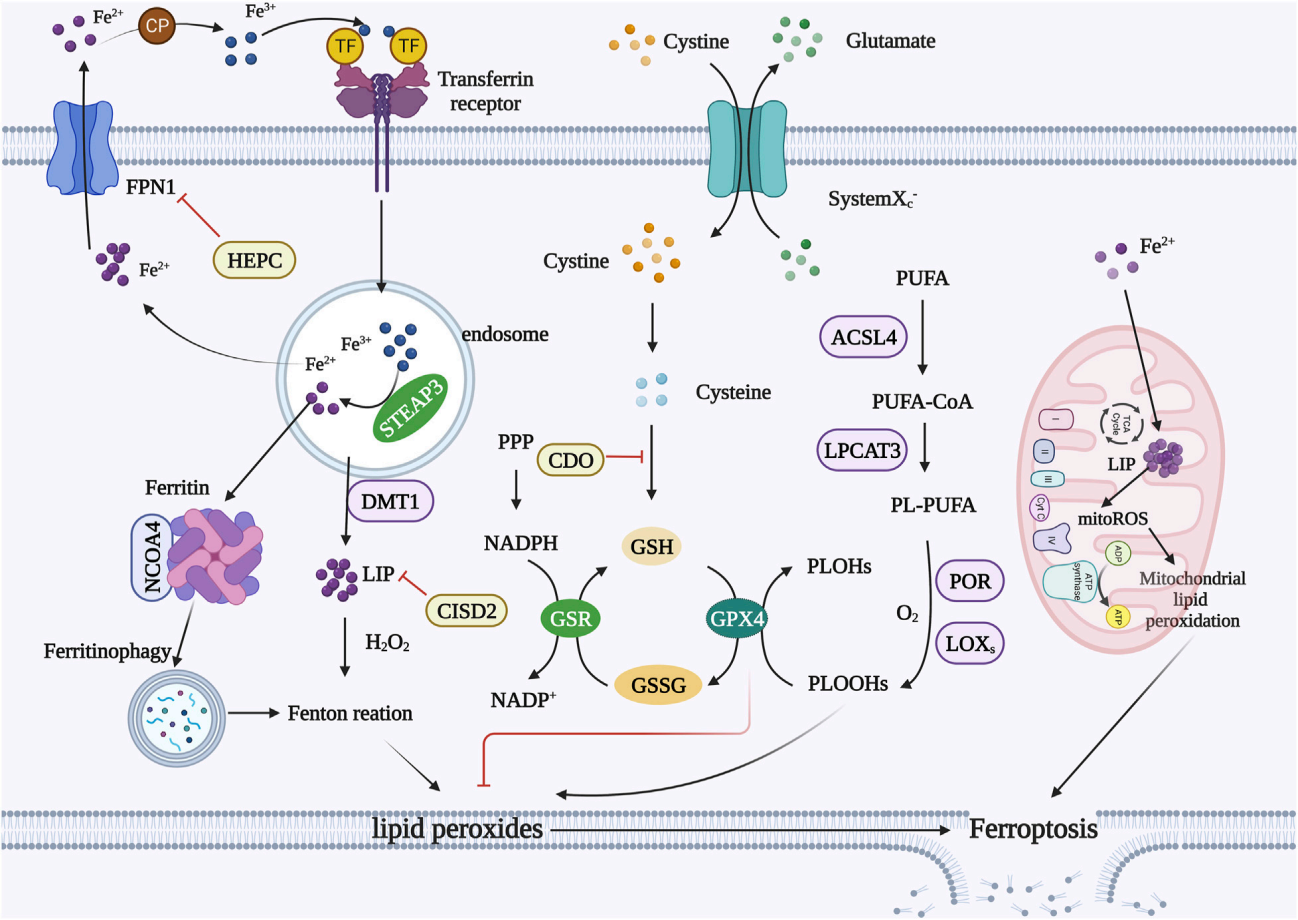
Mechanism of ferroptosis. **Iron metabolism**: extracellular Fe^2+^ can be oxidized to Fe^3+^, and Fe^3+^ binds to TF to form TF-Fe^3+^ and then binds to the TFR. Fe^3+^ is reduced to Fe^2+^ by STEAP3, and DMT1 transports reduced Fe^2+^ into the cytoplasm. Excess iron is stored in ferritin or the LIP. Free ferrous iron can cause lipid peroxidation through the Fenton reaction. FPN1 is the only channel through which intracellular Fe^2+^ is transported from cells. HEPC can bind to and degrade FPN1. CISD2 reduces free iron concentrations. NCOA4 promotes ferritin degradation while releasing free iron. **Amino acid metabolism**: Transport system x_c_^−^ exteriorizes intracellular glutamate while internalizing cystine, which is then reduced to cysteine in the cytoplasm. Cysteine is involved in the synthesis of GSH. GPX4 converts PLOOHs into PLOHs and converts GSH to GSSG. GPX4 can inhibit lipid peroxidation. **Lipid metabolism**: ACSL4 links free PUFAs to CoA to generate PUFA-CoA, which is then induced by LPCAT3 to form PL-PUFAs. PL-PUFAs can be further oxidized by LOXs to form lipid hydroperoxides and finally induce ferroptosis in cells. **Mitochondria:** Mitochondria also have an iron pool that causes a significant accumulation of mitoROS. MitoROS can react with PUFAs in the mitochondrial membrane, resulting in mitochondrial lipid peroxidation. **Abbreviations:** TFR, transferrin receptor; STEAP3, six transmembrane epithelial antigens of the prostate; DMT1, divalent metal transporter 1; LIP, labile iron pool; FPN1, ferroportin 1; HEPC, hepcidin; CISD2, CDGSH Iron Sulfur Domain 2; NCOA4, nuclear receptor coactivator 4; GSH, glutathione; GPX4, glutathione peroxidase 4; PLOOH, phospholipid hydroperoxide; PLOH, phospholipid-alcohol; GSSG, oxidized glutathione; ACSL4, long-chain lipid-CoA ligase 4; PUFA, polyunsaturated fatty acid; LPCAT3, lysophosphatidylcholine transferase 3; LOX, lipoxygenase; mitoROS, mitochondrial ROS.

Ferroptosis is mainly caused by the production of intracellular lipid ROS rather than their degradation. When the antioxidant capacity of cells decreases and lipid ROS accumulate, cell oxidative death can occur. Iron metabolism, amino acid metabolism, and lipid metabolism can control ferroptosis by regulating cellular redox status (Figure 1).

### 2.1 Iron metabolism

Extra iron produces ROS, which can cause ferroptosis. To avoid cell death and maintain redox balance, excess iron is stored in ferritin or the labile iron pool (Ma et al., 2016); in this way, the iron cycle regulates cellular iron homeostasis. Low levels of TFR reduce iron concentrations, thereby inhibiting ferroptosis (Shen et al., 2018). Heat shock protein β-1 (HSPB1) decreases the intracellular iron concentration by inhibiting TRF1 expression to inhibit ferroptosis (Li et al., 2020). Ferritin includes ferritin light chain (FTL) and ferritin heavy chain 1 (FTH1) (Bradley et al., 2016). FTH1 is a functional subunit that stores iron, has ferric oxidase activity, and can effectively reduce Fe^2+^ toxicity (Salatino et al., 2019). Therefore, FTH1 protects cancer cells from ferroptosis (Sun et al., 2016a; Du et al., 2019). Iron–sulfur clusters promote cancer cell avoidance of ferroptosis by reducing unstable iron pools. Studies have shown that CDGSH Iron Sulfur Domain 2 (CISD2) is highly expressed in HNSCC and may enhance cancer cell resistance to ferroptosis by increasing iron–sulfur clusters and reducing free iron concentration (Kim et al., 2018).

Although previous studies have shown that ferroptosis is not associated with autophagy, it can be promoted in many situations (Liu et al., 2020). Selective autophagy participates in ferroptosis by degrading antiferroptosis regulators (Hou et al., 2016; Yang et al., 2019). Several recent reviews have identified ferroptosis as autophagy-dependent cell death (Liu et al., 2020; Zhou et al., 2020). Nuclear receptor coactivator 4 binds directly to FTH1 and promotes ferritin degradation while releasing free iron, which is a consequence of iron autophagy (Dowdle et al., 2014; Mancias et al., 2014). Furthermore, overexpression of nuclear receptor coactivator 4 promotes ferroptosis (Fujimaki et al., 2019). The HPV16 oncoprotein can inhibit the host autophagy response, which may provide resistance to ferroptosis in HPV-positive HNSCC cells (Belleudi et al., 2015; Mattoscio et al., 2018). The autophagy-related 5 gene is highly expressed in high-risk HNSCC patients and is an important factor driving ferroptosis (Gao et al., 2016; Hou et al., 2016).

### 2.2 Amino acid metabolism

GPX4 can convert cytotoxic lipid hydroperoxide peroxides (L-OOH) in cells into nontoxic lipid alcohols (L-OH). Therefore, it is considered one of the most powerful antioxidant enzymes in the human body, regulating toxic lipid hydroperoxide and ferroptosis (Friedmann Angeli et al., 2014; Yang et al., 2014). When GSH (glutathione) is depleted, GPX4 is inactivated (Sato et al., 2018). NADPH is a GSH reductase that regulates ferroptosis by maintaining a reduced state of GSH. Transport system x_c_^−^ consists of solute carrier family 3 member 2 (SLC3A2) and solute carrier family 7 member 11 (SLC7A11), which are combined via disulfide bonds. This system exteriorizes intracellular glutamate while internalizing cystine, which is then reduced to cysteine in the cytoplasm. Cysteine is the rate-limiting precursor of GSH synthesis (Forman et al., 2009; Aquilano et al., 2014). Cysteine can be degraded to pyruvate and α-ketoglutarate (α-KG). Pyruvate is then metabolized to acetyl coenzyme A (acetyl-CoA), which cooperates with GSH to regulate ferroptosis (Shimada et al., 2016; Badgley et al., 2020). The acetyl-CoA derivative CoQ (ubiquinone) can be reduced to ubiquinol by oxidoreductase ferroptosis suppressor protein 1 (FSP1), and ubiquinol can prevent the proliferation of lipid peroxides (Bersuker et al., 2019; Doll et al., 2019). Therefore, the inhibition of CoA production by CoQ can inhibit ferroptosis. α-KG also induces ferroptosis during amino acid starvation.

Glutamine metabolism is closely related to ferroptosis regulation (Gao and Jiang, 2018). Tissue and plasma contain large amounts of glutamine, which can be decomposed into glutamate through the activity of glutaminase-2 (GLS2). Glutamate is a natural trigger of ferroptosis, and high glutamate concentrations inhibit the function of system x_c_^−^, resulting in ferroptosis (Imai et al., 2017; Floros et al., 2021). GLS2 is involved in regulating ferroptosis, and GLS2 is highly expressed during p53-dependent ferroptosis. Gao et al. showed that in the absence of cysteine, glutaminolysis promotes mitochondrial respiration and significant GPX4 oxidation of GSH, leading to rapid GSH depletion and strong ferroptosis. However, when glutaminolysis was inhibited, GSH oxidation slowed, and even in the absence of cysteine, ROS accumulation, lipid peroxidation, and ferroptosis were inhibited.

### 2.3 Lipid metabolism

The mass accumulation of lipid peroxides on cell membranes leads to membrane rupture and ferroptosis. Fatty acids are substrates for lipid peroxidation, and free polyunsaturated fatty acids (PUFAs) are substrates for the synthesis of lipid signal transduction mediators, which are easily oxidized to form lipid peroxides. However, free PUFAs must be esterified into membrane phospholipids and oxidized into lipid ROS to transmit ferroptosis signals (Kwon et al., 2015).

Lipid peroxidation can be divided into two mechanisms: non-enzymatic spontaneous autoxidation and enzyme-mediated processes catalyzed by several enzymes (McBean, 2012; Eling et al., 2015; Sun et al., 2016a; Hou et al., 2016; Tarangelo et al., 2018). Free ferrous iron reacts with hydrogen peroxide to form ferric iron and hydroxyl radicals in a non-enzymatic autooxidation process. This is called the Fenton reaction (Louandre et al., 2015; Yamaguchi et al., 2018). Hydroxyl radicals directly reacts with PUFAs to initiate lipid peroxidation (Ou et al., 2017). Long-chain lipid-CoA ligase 4 (ACSL4) links free PUFAs, such as arachidonic acid (AA) or epinephrine (AdA), to CoA to generate AA-CoA or AdA-CoA (Yuan et al., 2016; Doll et al., 2017; Kagan et al., 2017), which is then induced by LPCAT3 to esterify membrane phosphatidylethanolamine (PE) and form AA-PE or AdA-PE (Dixon et al., 2015; Doll et al., 2017). Decreased expression of ACSL4 and LPCAT3 reduces the accumulation of lipid peroxidation substrates in cells, thereby inhibiting ferroptosis. AA-PE or AdA-PE can be further oxidized by lipoxygenase (LOX) to form lipid hydroperoxides and finally induce ferroptosis in cells.

Mitochondrial iron is mainly involved in the biosynthesis of iron–sulfur clusters and erythroid synthesis (Stehling and Lill, 2013). Mitochondria also have an iron pool (Lv and Shang, 2018) that causes a significant accumulation of mitochondrial ROS (mitoROS) (Zorov et al., 2014). MitoROS can react with PUFAs in the mitochondrial membrane, resulting in mitochondrial lipid peroxidation and DNA damage (Barrera et al., 2016). Thus, when cystine starvation or erastin triggers GSH depletion, the mitochondrial tricarboxylic acid cycle also contributes to ferroptosis (Sabharwal and Schumacker, 2014). The notion that lipid peroxides accumulate in cell membranes and lead to cell rupture currently predominates (Urrutia et al., 2014). Nonetheless, there is increasing evidence that lipid peroxide accumulation in mitochondrial membranes can also trigger ferroptosis (Stockwell et al., 2017; Gao and Jiang, 2018).

## 3 Regulatory pathway of ferroptosis

### 3.1 GSH–GPX4 system

GPX4 can convert GSH to oxidized GSH and reduce phospholipid hydroperoxide (PLOOH) to nontoxic phospholipid-alcohol (PLOH), preventing the accumulation of lipid peroxides and degrading small molecules or slightly complex lipid peroxides (Thomas et al., 1990), thereby preventing ferroptosis. Cells with decreased GPX4 expression were more sensitive to ferroptosis, whereas cells with upregulated GPX4 expression inhibited ferroptosis. Previous data suggested that the antiferroptotic function of GPX4 was limited to the cytoplasm (Yant et al., 2003; Conrad et al., 2005; Imai et al., 2009; Liang et al., 2009; Schneider et al., 2009) and that its antiferroptotic function was absent in the mitochondria and nucleus (Imai et al., 2009). However, recent research suggests that mitochondrial GPX4 also plays a role in ferroptosis (Battaglia et al., 2020). Two pathways can inhibit GPX4 expression: direct inhibition by the small molecule RSL3 or indirect inhibition of GSH production through the cystine-glutamate anti-transporter system x_c_^−^ (Yang and Stockwell, 2016). SLC7A11, the upstream node of the GSH–GPX4 axis, is a key factor in ferroptosis (Chen et al., 2020). Studies have shown that cystine deficiency or SLC7A11-mediated blocking of cystine transport by erastin can lead to ferroptosis in many cancer cell lines (Dixon et al., 2012; Jiang et al., 2015; Zhang et al., 2018; Koppula et al., 2021). Conversely, SLC7A11 overexpression promotes GSH biosynthesis and resistance to ferrotosis (Dixon et al., 2012; Jiang et al., 2015; Zhang et al., 2018). Inhibition of SLC7A11 inhibits cysteine uptake and reduces GSH synthesis, and GSH depletion results in GPX4 inactivation, ROS accumulation, and ferroptosis in cancer cells (Yang et al., 2014; Xie et al., 2016; Angeli et al., 2017).

### 3.2 FSP1–CoQ system

GPX4 is the most important regulatory factor for inhibiting ferroptosis; however, in certain cell lines, GPX4 inactivation does not induce ferroptosis, suggesting that other inhibitory mechanisms exist (Bersuker et al., 2019). In the absence of GPX4, FSP1 can completely counteract lethal peroxidation and ferroptosis, suggesting that FSP1 acts independently of GPX4, which can inhibit lipid peroxidation and defend against ferroptosis (Bersuker et al., 2019; Doll et al., 2019). This FSP1 gene can complement the loss of GPX4 in cancer cells (Doll et al., 2019). FSP1 is an oxidoreductase that reduces ubiquinone (CoQ) to ubiquinol (CoQH2). Moreover, CoQH2 can sequester lipid peroxidation free radicals, thereby inhibiting ferroptosis by inhibiting lipid peroxidation. FSP1 inhibits ferroptosis by producing ubiquitin; however, its activity is restricted to the cell membrane. The plasma membrane localization of FSP1 is sufficient and necessary to inhibit ferroptosis (Bersuker et al., 2019; Doll et al., 2019). FSP1 expression has been positively correlated with resistance to ferroptosis in several cancer cell lines (Bersuker et al., 2019).

### 3.3 DHODH system

Dihydroorotate dehydrogenase (DHODH) is an antioxidant that captures free radicals and has antiferroptosis activity. Furthermore, it reportedly operates in parallel with mitochondrial GPX4 and thus could provide a new method for targeted cancer therapy (Mao et al., 2021). DHODH is an enzyme localized on the mitochondrial inner membrane (Mao et al., 2021). It can reduce CoQ to CoQH2 on the mitochondrial inner membrane (Garcia-Bermudez and Birsoy, 2021; Mao et al., 2021), which can compensate for the loss of GPX4, thereby decreasing mitochondrial lipid peroxidation. When GPX4 is deficient, DHODH is significantly increased, resulting in an increase in CoQH2, which can inhibit lipid peroxidation and prevent ferroptosis (Mao et al., 2021). The DHODH inhibitor induces low GPX4 tumor development. However, when combined with sulfasalazine, which has iron-inducing activity, this inhibitor can synergistically induce ferroptosis and inhibit high-GPX4 tumor development (Mao et al., 2021). This is the DHODH-mediated mitochondrial ferroptosis defense mechanism. Moreover, studies have shown that GPX4 acts on the mitochondria and cytoplasm, FSP1 acts on the plasma membrane, and DHODH acts on the mitochondria. Therefore, cytoplasmic GPX4 and FSP1 cannot control lipid peroxide production in the mitochondrial membrane. Similarly, DHODH cannot control the production of lipid peroxides on the plasma membrane.

### 3.4 GCH1–BH4 system

GTP cyclic hydrolase 1 (GCH1) is another important ferroptosis inhibitor (Kraft et al., 2020; Soula et al., 2020). GCH1 is the rate-limiting enzyme for tetrahydrobiopterin (BH4) synthesis (Thöny et al., 2000; Soula et al., 2020). BH4 is a free radical-trapping antioxidant involved in the production of neurotransmitters, aromatic amino acids, and nitric oxide (Mbah and Lyssiotis, 2022). BH4 can promote the formation of CoQ and block lipid peroxidation, thereby inhibiting ferroptosis (Soula et al., 2020). Additionally, this system for blocking ferroptosis is independent of GPX4. However, the role of the GCH1–BH4 system in head and neck cancer remains unclear.

**FIGURE 2:**
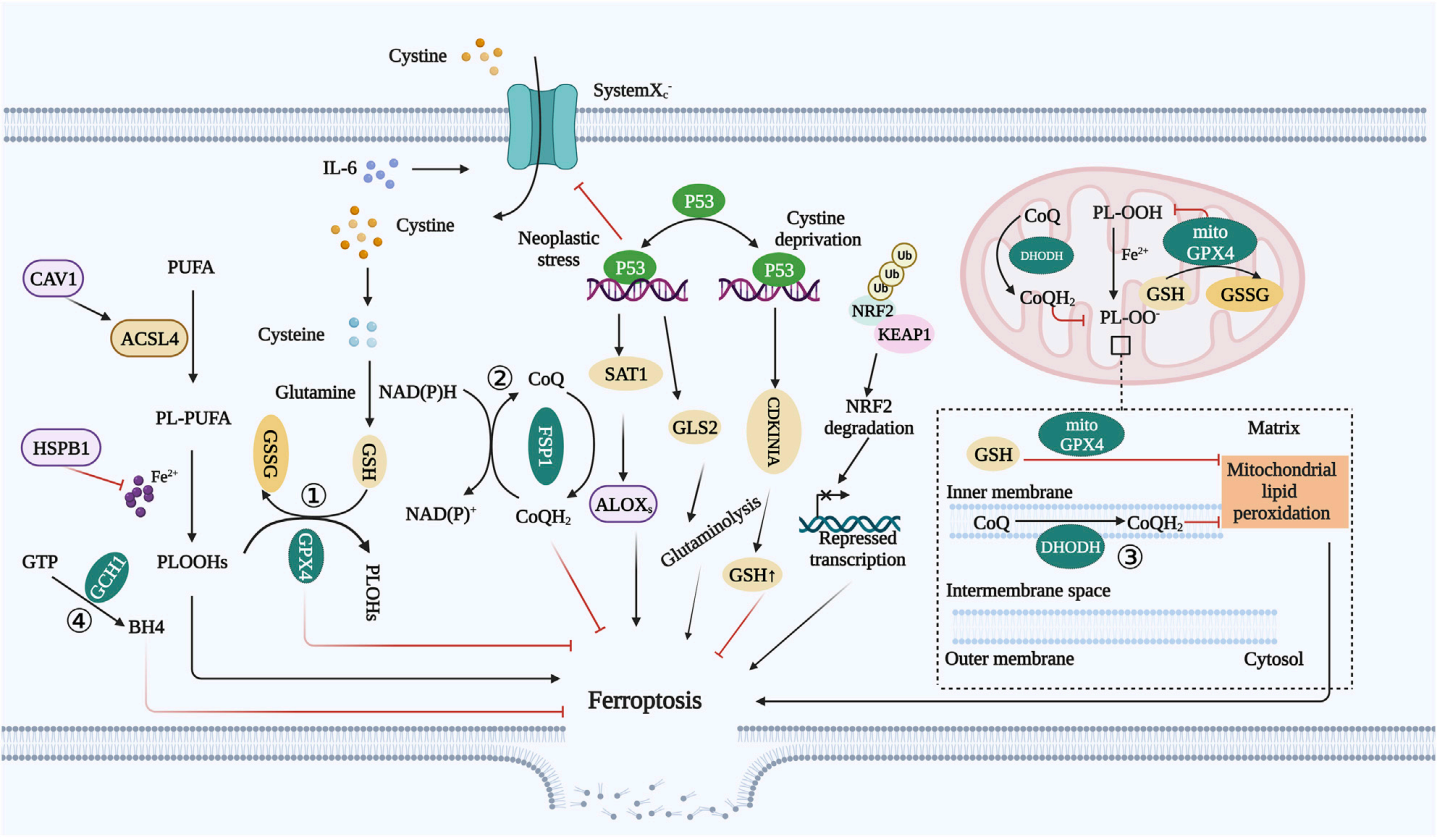
Regulatory pathway of ferroptosis. ① **GSH–GPX4 system:** Inhibition of SLC7A11 inhibits cysteine uptake and reduces GSH synthesis, and GSH depletion results in GPX4 inactivation, ROS accumulation, and ferroptosis in cancer cells. ② **FSP1–CoQ system:** FSP1 is an oxidoreductase that reduces CoQ to CoQH2. Moreover, CoQH2 can sequester lipid peroxidation free radicals, thereby inhibiting ferroptosis by inhibiting lipid peroxidation. ③ **DHODH system:** DHODH is an enzyme localized on the mitochondrial inner membrane. It can reduce CoQ to CoQH2 on the mitochondrial inner membrane, which can compensate for the loss of GPX4, thereby decreasing mitochondrial lipid peroxidation. ④ **GCH1–BH4 system:** GCH1 is the rate-limiting enzyme for BH4 synthesis. BH4 can promote the formation of CoQ and block lipid peroxidation, thereby inhibiting ferroptosis. **Abbreviations:** GSH, glutathione; GPX4, glutathione peroxidase 4; SLC7A11, solute carrier family 7 member 11; ROS, reactive oxygen species; FSP1, ferroptosis suppressor protein 1; CoQ, ubiquinone; CoQH2, ubiquinol; DHODH, dihydroorotate dehydrogenase; GCH1, GTP cyclic hydrolase 1; BH4, tetrahydrobiopterin; CAV1, caveolin-1; NRF2, nuclear factor erythroid 2-related factor 2; KEAP1, Kelch-like ECH-associated protein 1; IL-6, interleukin 6; HSPB1, heat shock protein B1.

The above four systems are the main mechanisms for regulating ferroptosis. Furthermore, there are regulatory factors that can modulate cell ferroptosis, with these systems at the center (Figure 2).

### 3.5 Ferroptosis regulatory factors

#### 3.5.1 Caveolin-1

Caveolin-1 (CAV1) is a membrane protein involved in cellular signal transduction and transport. Signal transduction related to CAV1 regulates lipid metabolism and causes cell death (Engelman et al., 1997; Xu et al., 2014). Studies on cancer cells with high CAV1 expression have shown that the expression of NOX1 and ACSL4 is increased, and the expression of FTH1 and GPX4 is decreased, which reduces sensitivity to ferroptosis. Conversely, CAV1 upregulation induces the accumulation of free Fe^2+^ in HNSCC cells, which accelerates tumor growth. Furthermore, downregulation of CAV1 promotes ferroptosis in cancer cells and inhibits tumor development (Lu et al., 2022).

#### 3.5.2 p53

p53 inhibits the expression of the SLC7A11 subunit by interacting with ubiquitin-specific processing protease 7 (usP7) or directly interacting with the SLC7A11 promoter, inhibiting the activity of system x_c_^−^ and causing ferroptosis in tumor cells (Xie et al., 2017). High p53 expression can upregulate Glutaminase-2 (GLS2) transcription, increase GSH synthesis, lead to ferroptosis in tumor cells, and inhibit tumor cell growth (Kang et al., 2019). p53 can also induce ferroptosis by regulating the expression of other metabolic targets (Jennis et al., 2016; Ou et al., 2016; Zhang et al., 2017). Certain mutant forms of p53, such as the acylation-deficient p53 mutant (TP53-3 KR), are unable to induce apoptosis but can inhibit tumor growth *in vivo* by promoting ferroptosis (Wang et al., 2016). Previous studies have shown that recombinant human p53 adenovirus promotes radiation sensitivity in recurrent nasopharyngeal carcinoma (Ma et al., 2017). Interestingly, p53 can activate cyclin-dependent kinase inhibitor 1A (CDKN1A)/p21 or inhibit dipeptidyl peptidase 4 (DPP4) to prevent ferroptosis under specific conditions (Xie et al., 2017). Therefore, depending on the cellular context, p53 may play a dual role in regulating ferroptosis (Xie et al., 2017; Tarangelo et al., 2018).

#### 3.5.3 NRF2

Nuclear factor erythroid 2-related factor 2 (NRF2) is the most important antioxidant regulator involved in lipid peroxidation (Ingold et al., 2018). Almost all ferroptosis-related genes are transcriptionally regulated by NRF2 through the GSH–GPX4 system, NADPH regeneration, and iron metabolism-regulated genes, including FTL/FTH1, FPN, heme oxygenase-1 (HO-1), and solute carrier family 48 member 1 (SLC48A1) (Sun et al., 2016a; Kerins and Ooi, 2018). NRF2 also indirectly regulates lipid peroxidation, and reducing NRF2 levels can promote ROS production (Zhang et al., 2017) NRF2 activation results in cancer cell resistance to ferroptosis (Sun et al., 2016a; Fan et al., 2017; Roh et al., 2017). Kelch-like ECH-associated protein 1 (KEAP1) is an endogenous inhibitor of NRF2 that regulates NRF2 expression through the ubiquitin-proteasome pathway (Seibt et al., 2019). Under oxidative stress conditions, KEAP1 mutations and NRF2 cannot be isolated or degraded. NRF2 is transferred to the nucleus, recognizes and binds to antioxidant response elements (AREs), and regulates ferroptosis-related genes, thereby regulating ferroptosis (Bellezza et al., 2018; Rojo de la Vega et al., 2018). KEAP1 levels higher than those in normal mucosa can be detected in HNSCC. Inhibition of NRF2 or KEAP1 gene transfection can cause overexpression of NRF2 and enhance the resistance of HN3 cells to RSL3, an inhibitor of GPX4. Conversely, NRF2 inhibition sensitizes HN3 cells to RSL3 (Shin et al., 2018). Thus, NRF2 inhibition can eliminate cancer cell resistance to ferroptosis. NRF2 is highly expressed in many cancers (Rojo de la Vega et al., 2018; Dodson et al., 2019; Anandhan et al., 2020), including HNSCC cells, and *NRF2* knockout can reduce GSH levels in HNSCC cells (Wang et al., 2017; Ramesh et al., 2020).

#### 3.5.4 IL-6

The high expression of Interleukin (IL)-6 is closely related to tumor development and lymph node metastasis in patients with HNSCC (Chang et al., 2013). The upregulation of IL-6 has been reported to promote the malignant transformation of leukoplakia into cancer (Zhang et al., 2013; Kaur and Jacobs, 2015; Babiuch et al., 2020). Studies have shown that IL-6 can transcriptionally activate the expression of SLC7A11 through the Janus kinase (JAK) 2/signal transducer and activator of transcription (STAT)3 signaling pathway to resist ferroptosis and promote tumor development in HNSCC.

#### 3.5.5 HSPB1

Heat shock proteins are overexpressed in cancer cells and play important roles in cancer cell invasion, proliferation, and angiogenesis. HSPB1 is a member of the heat shock protein family and is a major factor controlling heat shock protein expression (Wu, 1995). HSPB1 phosphorylation inhibits ferroptosis by inhibiting iron accumulation and the production of lipid ROS (Sun et al., 2015). Inhibition of HSPB1 phosphorylation enhances erastin-induced ferroptosis and increases the anticancer activity of erastin *in vivo* (Sun et al., 2015). However, the effect of HSPB1 on ferroptosis in HNSCC cells remains unclear.

#### 3.5.6 ncRNAs

Noncoding RNAs (ncRNAs) can affect gene expression and disease progression, making them new targets for drug treatment. Various ncRNAs have been identified, including small noncoding RNAs (miRNAs), circular RNAs (circRNAs), long noncoding RNAs (lncRNAs), small nuclear RNAs (snRNAs), piwi-interacting RNAs (piRNAs), small nucleolar RNAs (snoRNAs), ribosomal RNAs (rRNAs), and transfer RNAs (tRNAs). Among them, miRNAs, circRNAs, and lncRNAs are regulatory ncRNAs (Chen, 2016; Wu et al., 2017; Yao et al., 2019). Regulatory ncRNAs have been widely studied in recent years and play key roles in HNSCC proliferation and metastasis (Wang et al., 2018; Wang et al., 2020) (Table 1). Related studies have demonstrated that several ncRNAs are abnormal in radiosensitive or radioresistant HNSCC tissues (Qu et al., 2015a; Qu et al., 2015b; de Jong et al., 2015; Suh et al., 2015; Xu et al., 2015; Gao et al., 2017; Hess et al., 2017; Vahabi et al., 2019; Pasi et al., 2020). The differential expression of certain ncRNAs can significantly affect the response of HNSCC cells to chemotherapy or radiotherapy, suggesting that ncRNAs can regulate the sensitivity of cancer cells to treatment. Extensive literature suggests that ncRNAs play an important role in the regulation of ferroptosis in HNSCC.

**TABLE 1:** Ferroptosis-associated ncRNAs in HNSCC.

Category	Molecular	Regulation Effect on Ferroptosis in HNSCC	Mechanism	Associated Type of HNSCC	References
miRNA	miR-214	↓	regulating LTF	nasopharyngeal carcinoma	Deng et al. (2013a)
	miR-107	↑	regulate protein kinaseC by regulating TFR	HNSCC	Datta et al. (2012)
	miR-148a	↑	regulate protein kinaseC by regulating TFR	HNSCC	Datta et al. (2012)
	pre-miR-107	↑		HNSCC	Piao et al. (2012)
	miR-210	↓	inhibiting TFR expression	OSCC	Yoshioka et al. (2012), Gee et al. (2014)
	miR-153	↑	downregulating NRF2	HNSCC	Shah et al. (2015), Wang et al. (2016b), Chen et al. (2019a)
	miR -125b	↑	downregulating NRF2	HNSCC	Shah et al. (2015), Wang et al. (2016b), Chen et al. (2019a)
	miR -17–92	↓	downregulating ACSL4 expression	HNSCC	Xiao et al. (2019)
	miR-125b-5p	↑	Inhibit SLC7A11	OSCC	Yu et al. (2021)
	miR-137	↑	acts on SLC7A11 to decrease glutamine uptake	HNSCC	Luo et al. (2018)
	miR -4715−3p	↑	inhibit GPX4 expression	HNSCC	Luo et al. (2018)
	miR-372	↑	activation of thep53 signaling pathway	nasopharyngeal carcinoma	Wang et al. (2019a)
circRNA	circFNDC3B	↓	bea competing endogenous RNAs (ceRNA) of miR-520d-5p to increase the expression of SLC7A11	OSCC	Yang et al. (2021)
LncRNA	LncRNA TINCR	↓	regulating acetyl−CoA metabolism	nasopharyngeal carcinoma	Zheng et al. (2020)
	LncRNA HOTAIR	↓	regulating the *de novo* synthesis of fatty acids	nasopharyngeal carcinoma	Ma et al. (2017b)
	LINC00669	↑	downregulating SLC7A11 expression	nasopharyngeal carcinoma	Saint-Germain et al. (2017)

LTF, lactotransferrin; TFR, transferrin receptor; NRF2, Nuclear factor erythroid 2−related factor2; ACSL4,long-chain lipid−CoA, ligase4; SLC7A11, solute carrier family7 member11; GPX4, peroxidase4.

In addition, many miRNAs play a role in the regulation of ferroptosis. For example, miR-214 is upregulated in nasopharyngeal carcinoma tissues, especially in nasopharyngeal carcinoma tissues, with a tendency to metastasize. Additionally, this miRNA can regulate the ferroptosis of nasopharyngeal carcinoma cells by regulating lactotransferrin (LTF) (Deng et al., 2013a). miR-107 and miR-148a can regulate protein kinase C by regulating TFR to exert an HNSCC tumor-suppressor effect (Datta et al., 2012). Lipid-based nanoparticle delivery of pre-miR-107 can reduce HNSCC progression (Piao et al., 2012). miR-210 inhibits ferroptosis in oral squamous cell carcinoma (OSCC) cells by inhibiting TFR expression (Yoshioka et al., 2012; Gee et al., 2014). miR-153 and miR-125b promote ferroptosis in HNSCC cells by downregulating NRF2 (Shah et al., 2015; Wang et al., 2016; Chen et al., 2019). miR-17-92 can reduce the accumulation of lipid peroxides and protect cells from ferroptosis by downregulating ACSL4 expression (Xiao et al., 2019). miR-125b-5p is underexpressed, negatively regulates SLC7A11 expression in OSCC, promotes ferroptosis, and inhibits cell proliferation (Yu et al., 2021). miR-137 acts on SLC1A5 to decrease glutamine uptake and induce ferroptosis (Luo et al., 2018). miR -4715-3p can inhibit GPX4 expression to induce ferroptosis (Gomaa et al., 2019). Finally, Wang et al. reported that overexpression of miR-372 and activation of the p53 signaling pathway enhance the radiosensitivity of nasopharyngeal carcinoma (Wang et al., 2019).

CircRNA molecules have a closed circular structure and are not affected by RNA exonucleases, displaying stable expression. Their dysregulation can lead to the development of many types of cancer (Hentze and Preiss, 2013). CircRNAs are also involved in ferroptosis regulation. They can competitively bind to miRNAs and function as miRNA sponges (Salmena et al., 2011; Memczak et al., 2013). For example, circRNA cIARS regulates ferroptosis by suppressing the RNA-binding protein alkB homolog 5 (ALKBH5)-mediated autophagy inhibition in hepatocellular carcinoma cells (Liu et al., 2020). In OSCC, circFNDC3B can be a ceRNA of miR-520d-5p that increases the expression of SLC7A11 (Yang et al., 2021).

The role of lncRNAs in gene regulation has received increasing attention (Ulitsky and Bartel, 2013). LncRNAs can act as both regulatory molecules for transcription factors and sponges for miRNAs in the cytoplasm (Salmena et al., 2011). Many lncRNAs inhibit ferroptosis in cancer cells. For example, lncRNA LINC00336 binds to the embryonic lethal abnormal visual system (ELAV)-like RNA binding protein 1 (ELAVL1) and inhibits ferroptosis by reducing intracellular iron content and lipid ROS levels (Wang et al., 2019). The lncRNA P53RRA activates p53 through Ras GTPase activator protein binding protein 1 (G3BP1), which induces ferroptosis by affecting multiple pathways (Mao et al., 2018). The lncRNA ZNFX1 antisense RNA 1 (ZFAS1) inhibits ferroptosis by downregulating solute carrier family 38 member 1 (SLC38A1) as a ceRNA of miR-150-5p (Yang et al., 2020). Terminal differentiation-induced noncoding RNA (TINCR) promotes nasopharyngeal carcinoma progression and chemoresistance by regulating acetyl-CoA metabolism (Zheng et al., 2020). HOX antisense intergenic RNA (HOTAIR) inhibits ferroptosis in nasopharyngeal carcinoma cells by regulating the *de novo* synthesis of fatty acids (Ma et al., 2017). LINC00669 protects the suppressor of cytokine signaling 1 (SOCS1) from ubiquitination, which induces ferroptosis by downregulating SLC7A11 expression via p53 in nasopharyngeal carcinoma (Saint-Germain et al., 2017).

Ferroptosis-associated lncRNAs play an important role in predicting HNSCC prognosis. Multiple ferroptosis-related lncRNAs, such as LINC01963, are independent predictors of HNSCC (Tang et al., 2021). Guo et al. found that six ferroptosis-related lncRNAs might promote tumorigenesis in HNSCC (Guo Q. et al., 2021). In addition, six ferroptosis-related lncRNAs have been suggested as predictors (Jiang et al., 2021). Furthermore, there were nine ferroptosis-related-lncRNAs with prognostic ability for patients with HNSCC, among which AC010894.2 was associated with poor prognosis, and the other eight predicted a favorable prognosis (Lu et al., 2022). Prognostic models of eight ferroptosis-related lncRNAs in patients with OSCC have been described. Among these, AC021087.4, HOTARM1, AC090246.1, Alstrom syndrome gene intronic script 1 (ALMS1-IT1), and AC099850.3 were associated with poor prognosis. Conversely, AL512274.1, myocardial infarction-associated transcript, and StAR-related lipid transfer protein 4 antisense RNA 1 (STARD4-AS1) had favorable prognostic effects (Li et al., 2021).

## 4 Treatment

In recent years, the application of several therapeutic methods has accelerated tumor treatment. Despite the continuous improvement of basic and clinical research levels, the treatment effect of patients is still poor, with recurrence, metastasis, and ultimately death. The main barrier for treating patients with recurrent, or metastatic HNSCC is resistance to chemotherapeutic drugs. Hence, there is an urgent need to find new therapeutic strategies to effectively overcome drug resistance. By studying the mechanism of ferroptosis and regulating the signal axis of ferroptosis, a new therapeutic strategy can be formed to induce ferroptosis in HNSCC, including drug therapy, radiation therapy, immunotherapy, nano therapy and so on. In particular, the combined use of multiple therapeutic means provides a new target for the treatment of HNSCC.

### 4.1 Drug therapy

Many studies have found that ferroptosis can reverse drug resistance of HNSCC cells or enhance cell sensitivity to chemotherapeutic drugs (Roh et al., 2016; Ye et al., 2019). The discovery of ferroptosis inducing drugs that can kill resistant cancer cells has opened up new therapeutic areas for cancer treatment. People are constantly searching for the target signals and molecules that can induce ferroptosis (Chang et al., 2013; Ramesh et al., 2020), and various ferroptosis regulators have been discovered, including ferroptosis inducers and ferroptosis inhibitors. Among them, ferroptosis inducers play a role in inhibiting the development of cancer, especially drug-resistant cancer (Table 2). At present, there are few studies on therapeutic drugs for HNSCC. In this paper, the ferroptosis regulators associated with HNSCC or commonly used in other epithelial tumors are listed as follows.

**TABLE 2:** Inducers of ferroptosis.

Induction mode	Agent	Functional mechanism	References
System xc− Inhibition	Erastin	Inhibit SLC7A11,preventing cysteine-dependent GSH synthesis, and inactivating GPX4	Sato et al. (2018)
	Sulfasalazine	inhibition of system xc− transporter	Ma et al. (2015)
	Sorafenib	blocks the activity of system xc− and inhibits GSH synthesis	Louandre et al. (2013), Lachaier et al. (2014)
GSH inhibition	Olaparib	inhibit SLC7A11	Hong et al. (2021)
Iron activation	Cisplatin	Reduce GSH and inactivate GPX4	Guo et al. (2018)
	artemisinin	Induction of ferritinophagy in ferritin leading to increased intracellular iron levels	Du et al. (2019)
GPX4 inhibition	RSL3	inactivating GPX4 and inducing significant ROS production	Sui et al. (2018), Vučković et al. (2020)
	FIN56	regulating GPX4 degradation	Shimada et al. (2016)
HMGCR inhibition	Statins	control the synthesis of GPX4 by depleting CoQ10	Chen and Galluzzi (2018)

SLC7A11, solute carrier family 7 member 11; GSH, glutathione; GPX4, peroxidase 4; RSL3, ras-selective lethal small molecule 3; ROS, reactive oxygen species; HMGCR, hydroxymethylglutaryl CoA reductase.

#### 4.1.1 Ferroptosis inducers

Ferroptosis inducers can be divided into 4 types according to their target and mechanism of action (Table 2): system xc− inhibition, GSH inhibition, iron activation and GPX4 inhibition.

##### 4.1.1.1 System xc− inhibition

Erastin is the first known drug to induce ferroptosis. It can regulate a variety of molecules such as SLC7A11 (Sato et al., 2018). This drug inhibits cystine transmission inside and outside the cell, resulting in GPX4 inactivation, GSH depletion, and subsequent ferroptosis. Erastin can also open voltage-dependent anion channels to alter mitochondrial membrane permeability and increase ROS production, thereby causing ferroptosis (Fang and Maldonado, 2018). Erastin combined can enhance the activity of antitumor drugs and exert a significant clinical effect in tumor treatment (Louandre et al., 2013; Ma et al., 2015; Roh et al., 2016; Roh et al., 2017; Chen et al., 2019).

The SLC7A11 inhibitor sulfadiazine promotes cellular ferroptosis by reducing GSH and improves the antitumor effects of cisplatin by enhancing drug transport (Ma et al., 2015). Cysteine starvation or SLC7A11 inhibition induces ferroptosis and reverses cisplatin resistance in HNSCC cells. Thus, sulfadiazine is highly effective and has been approved as an anticancer drug. In OSC19 and HSC-4 cells, sulfadiazine can significantly reduce the levels of intracellular cysteine and GSH. In addition, inhibition of GPX4 and system x_c_^−^ can lead to an abnormal increase in ROS levels, causing drug-resistant cancer cells to become sensitive to ferroptosis (Yoshikawa et al., 2013; Liu et al., 2017) and promoting cancer cell death (Doxsee et al., 2007; Guan et al., 2009; Xie et al., 2016). However, resistance to sulfadiazine may also occur. Studies have shown that aldehyde dehydrogenase 3 family member A1 (ALDH3A1) is overexpressed in sulfadiazine-resistant HNSCC cells and plays an important role in protecting cells from lipid peroxidation. This resistance can be reversed by the ALDH inhibitor dyclonine (Okazaki et al., 2018). Combined treatment with sulfadiazine and dyclonine significantly increased intracellular ROS levels in HSC-4 cells and promoted ferroptosis in cancer cells. Cysteine-deficient cancer cells may manifest as weakened lipid peroxidation, insensitivity to sulfadiazine, and leading to drug resistance. However, dyclonine inhibits antioxidant systems independently of SLC7A11, and increase the sensitivity of cancer cells to sulfadiazine. Furthermore, the inhibition of CISD2 can increase mitochondrial iron accumulation and lipid ROS production and overcome the resistance of HNSCC to sulfadiazine-induced ferroptosis (Kim et al., 2018).

Sorafenib is also a ferroptosis inducer that has been approved as an anticancer drug. Sorafenib has been reported to have pharmacological effects similar to erastin. This drug blocks the activity of system x_c_^−^ and inhibits the synthesis of GSH, and leads to significant accumulation of intracellular ROS and ferroptosis in cancer cells (Louandre et al., 2013; Lachaier et al., 2014). However, tumors can become prone to develop resistance to sorafenib, resulting in poor prognosis. Sorafenib resistance is caused by the arrest of ferroptosis in cancer cells and can be restored *in vivo* by combining metallothionein-1G (MT-1G) inhibitors (Sun et al., 2016b; Jiang et al., 2021). Metallothionein, a cysteine-rich protein, plays a key role in oxidative stress responses. Upregulation of MT-1G, which is a functional isoform of metallothionein-1, inhibits sorafenib-induced ferroptosis by inhibiting GSH depletion and lipid peroxidation. Inhibition of MT-1G expression can enhance the anticancer effect of sorafenib by promoting ferroptosis (Sun et al., 2016b).

Olaparib is an inhibitor of poly (ADP-ribose) polymerase (PARP). SLC7A11 is the catalytic subunit of system x_c_^−^, which regulates the ferroptosis defense system by increasing the cysteine supply and GSH biosynthesis. Olaparib can inhibit SLC7A11, thereby inhibiting GSH synthesis by promoting ferroptosis (Hong et al., 2021).

##### 4.1.1.2 GSH inhibition

Cisplatin is the first-line treatment for cancer and a commonly used drug for HNSCC (Galluzzi et al., 2014). This drug is also an inducer of ferroptosis. Cisplatin triggers ferroptosis via oxidative stress and lipid peroxidation (Griso et al., 2022). Cisplatin causes GPX4 inactivation and GSH downregulation in HNSCC cells, resulting in ferroptosis. Cisplatin combined with erastin significantly improves antitumor efficacy (Guo et al., 2018). PRLX93936 is a compound similar to erastin. Combining cisplatin and PRLX93936 induces lipid peroxidation and excess iron production, ultimately promoting ferroptosis (Liang et al., 2021). However, in clinical treatment, cisplatin is usually associated with toxicities in various organs and drug resistance, which is the main reason for its limited clinical application (Galluzzi et al., 2012).

##### 4.1.1.3 Iron activation

In recent years, artemisinin and its derivatives-dihydroartemisinin (DHA) and artesunate-have been widely used in anti-tumor research. Artemisinin can promote ferroptosis in HNSCC by inhibiting the KEAP1-NRF2-ARE pathway (Roh et al., 2017). DHA promotes autophagic degradation of ferroptosis by regulating the AMP-activated protein kinase (AMPK)/mechanistic target of rapamycin (mTOR)/p70S6k signaling pathway, leading to an increase in iron pools and ROS accumulation, inducing ferroptosis (Du et al., 2019). In addition, DHA promotes GPX4 inhibition-mediated ferroptosis in cancer cells resistant to ferroptosis (Chen et al., 2020). Lin et al. showed that DHA can induce ferroptosis in head and neck cancer cells, leading to cell cycle arrest (Lin et al., 2016), which illustrates the key role of ferroptosis in HNSCC. The anti-tumor effect of artesunate induces siderocytosis and ROS accumulation in an iron-dependent manner, causing ferroptosis. Additionally, it can selectively kill head and neck cancer cells (Ooko et al., 2015). Itraconazole can reduce the viability of nasopharyngeal carcinoma cells by increasing intracellular iron concentrations and lipid peroxide in the lysosomes (Xu et al., 2021).

##### 4.1.1.4 GPX4 inhibition

RSL3 potently induces ferroptosis by inactivating GPX4 and inducing significant ROS production (Yang et al., 2014; Sui et al., 2018; Vučković et al., 2020). RSL3, combined with a low concentration of PTX, can upregulate p53 protein expression and induce ferroptosis in hypopharyngeal squamous cell carcinoma cells (Ye et al., 2019). FIN56 induces ferroptosis by regulating GPX4 degradation, binds to squalene synthase, and activates it to inhibit CoA and promote ferroptosis (Shimada et al., 2016). 1,2-dioxoquinoline is a peroxide that induces ferroptosis by affecting the iron concentration and inactivating GPX4 (Abrams et al., 2016). Methotrexate, a dihydrofolate reductase (DHFR) inhibitor, synergistically kills cancer cells with GPX4 inhibitors (Soula et al., 2020).

##### 4.1.1.5 HMGCR inhibition

Hydroxymethyl glutaryl CoA reductase (HMGCR) can control the synthesis of GPX4 by depleting CoQ and sensitize cells to ferroptosis. Therefore, statins may also contribute to ferroptosis, as they inhibit HMG-CoA (Chen and Galluzzi, 2018). The use of statins has reduced the incidence of many types of cancer, and this review highlights new prospects for statins in cancer treatment (Pisanti et al., 2014).

#### 4.1.2 Ferroptosis inhibitors

There are two common drugs for ferroptosis inhibitors. Ferrostatin-1 (Fer-1) is a specific inhibitor of ferroptosis, which can reduce ROS and lipid peroxidation, and protect HT-22 cells from glutamate-induced oxidative toxicity and ferroptosis (Hu et al., 2019; Chu et al., 2020). Zileuton is a 5-LOX inhibitor that inhibits ferroptosis by barring the production of cellular ROS. It can protect neurons from oxidative stress caused by glutamate (Liu et al., 2015).

### 4.2 Radiation therapy

Radiation-induced potent ferroptosis is an important mechanism of radiation therapy (RT) against cancer (Lang et al., 2019; Lei et al., 2020; Ye et al., 2020). Clinically, RT combined with chemotherapy or immunotherapy is often required to kill cancer cells. RT can damage DNA and inhibit GSH production, thereby promoting GPX4-mediated ferroptosis (Kirtonia et al., 2020). RT also upregulates ACSL4 expression to promote PUFA-PL biosynthesis. In addition, RT can induce oxidative stress in cancer cells, leading to lipid peroxidation and cellular ferroptosis (Lang et al., 2019; Lei et al., 2020; Ye et al., 2020; Lei et al., 2021). LTF may negatively regulate the occurrence and metastasis of nasopharyngeal carcinoma (Zhang et al., 2015). Studies have shown that the mitogen-activated protein kinase (MAPK)/AKT pathway is important for tumor radiosensitization. Moreover, LTF overexpression can inhibit the proliferation of nasopharyngeal carcinoma cells by regulating the MAPK/AKT pathway. Therefore, radiotherapy can treat nasopharyngeal carcinoma by regulating the LTF level (Zhou et al., 2008; Zhang et al., 2011; Deng et al., 2013b; Song et al., 2019). Taken together, these results suggest that RT can abolish the radioresistance of tumor cells by inducing ferroptosis, which has important therapeutic value. Certain HNSCC cells are insensitive to other types of cell death, including autophagy and apoptosis. Therefore, the induction of ferroptosis may be an optimal strategy for RT in HNSCC. Nonetheless, adverse RT events, such as the death of granulocyte-macrophage hematopoietic progenitor cells and pulmonary fibrosis, are associated with ferroptosis (Li et al., 2019; Zhang et al., 2020). Therefore, radiation-induced hematopoietic damage may be ameliorated by inhibiting ferroptosis.

### 4.3 Immunotherapy

Tumor immunotherapy exerts antitumor effects by enhancing the effects of CD8^+^ T cells in the tumor microenvironment (TME) (Abbott and Ustoyev, 2019; von Witzleben et al., 2020; Ruffin et al., 2021). Immunotherapy stimulates the CD8^+^ T cell population to release interferon (IFN), which can reduce system x_c_^−^ expression and GSH synthesis, thereby promoting lipid peroxidation and inducing ferroptosis (Wang et al., 2019). Tumor-associated macrophages are the predominant phagocytic cells in the TME, and their depletion suppresses immunostimulatory functions. M1 macrophages are more easily activated than M2 macrophages and produce IFN (Mills et al., 2000). Therefore, repolarization of M2 to M1 macrophages in the TME may be a more effective method to improve the efficacy of immunotherapy. This may be achieved by increasing SOCS1 expression or reducing FTH1 expression to induce ferroptosis in HNSCC (Goossens et al., 2019). However, owing to the heavy physical barrier formed by fibroblasts and the extracellular matrix in the TME, drug delivery is obstructed, thereby hindering the efficacy of immunotherapy. Combination therapy using immune checkpoint inhibitors and ferroptosis inducers has broad application prospects. In HNSCC, the use of thiazoxide selectively induces oxidative stress, leading to ferroptosis and thereby reducing the number of stem-like cells with high expression of CD44v (CD44vhigh) without affecting cells with low expression or no detectable CD44v (CD44vlow-neg) (Dolma et al., 2003). In HNSCC and gastric cancer, CD44vhigh stem-like tumor cells may be more dependent on the CD44v-xCT axis for survival (Yoshikawa et al., 2013). T cell-induced ferroptosis in cancer cells is a newly discovered anticancer mechanism. The combination of a cystinase inhibitor with immunotherapy can synergistically strengthen T cell-mediated antitumor immunity and trigger ferroptosis in cancer cells (Lei et al., 2020). The combination of tryptophan deficiency and immune checkpoint blockade can also induce ferroptosis by enhancing T cell-mediated antitumor immunity (Wang et al., 2019).

### 4.4 Nanotherapy

In addition, nanodrug development and exosome technology can increase intracellular free iron or ROS and induce ferroptosis. Nanomaterials can integrate multiple functions to exert therapeutic effects via microcarriers. Polystyrene nanoparticles can effectively reduce ROS and inhibit ferroptosis by triggering transcription factor EB (TFEB) nuclear translocation and lysosomal stress (Li et al., 2019). Nanoparticle-packaged formulations can precisely target cancer cells. The modified nanoparticles have been co-loaded with cyclodextrin (sk-cd) iron and transforming growth factor (TGF)-AKT (SB431542), which can activate apoptosis by increasing intracellular ROS levels (Zhang et al., 2021). Sun et al. developed a ROS-responsive nanoparticle (NP-sfb/ce6) co-loaded with sorafenib and chlorin e6 to enhance the antitumor effect. Upon irradiation at 660 nm, chlorin e6 generated ROS and disrupted the nanoparticles, thereby promoting the release of sorafenib from NP-sfb/ce6. Sorafenib released by low-dose irradiation reduces tumor progression by inducing powerful antitumor immunity in T cells, disrupting the interaction between CD8^+^ T cells and a variety of immunosuppressive cells and reshaping the TME (Sun et al., 2020). Ce6-erastin nanoparticles plus photodynamic therapy significantly reduce the expression of SLC7A11, which could be a new treatment for OSCC. New materials, such as zero-valent iron nanoparticles, are resistant to OSCC treatment, in part through ferroptosis (Huang et al., 2019). Furthermore, exosomes can be combined with nanomaterials for antitumor therapies. Various platinum-free iron oxide nanoparticles are absorbed and degraded through exosomal channels in the acidic environment of cancer and then release intracellular iron, which enhances ROS production and leads to ferroptosis (Ma et al., 2017).

### 4.5 Comprehensive treatment

Traditional tumor treatments, such as radiation and chemotherapy, often induce resistance, leading to poor results. Nevertheless, radiation or chemotherapy combined with immunotherapy often improves the results of cancer treatment. Radioresistance is the primary factor leading to radiotherapy failure, tumor recurrence, and metastasis. Studies have shown that RT induces KEAP1 mutations and upregulates SLC7A11 expression by regulating NRF2, triggering resistance to ferrotosis (Lei et al., 2020). Immunotherapy-activated CD8^+^ T cells increase ROS production and promote apoptosis by releasing IFN, rendering tumor cells sensitive to RT.

Therefore, ferroptosis is a new mechanism for promoting the synergistic effects of immunity, radiotherapy, and chemotherapy. Many studies have shown that chemotherapy and radiotherapy can inhibit tumor development by inducing apoptosis in cancer cells (Friedmann Angeli et al., 2019; Chen et al., 2021a). However, the association between hyperferritinemia and cancer immunotherapy has been poorly researched, and only a few ferroptosis-related genes have been associated with prognosis (Chen et al., 2021b; Wang et al., 2021). These findings elucidate the relationship between immunotherapy and ferroptosis and lay a theoretical foundation for the study of ferroptosis inducers and immunotherapy in the combinatorial treatment of tumors.

## 5 Conclusions and outlook

Here, we systematically review the specific molecular mechanisms of ferroptosis, which involve a variety of biomolecules and signaling pathways. By regulating the expression of these biomolecules and signaling pathways, it is possible to alter the sensitivity of cancer cells to ferroptosis, thereby inhibiting tumor development. Through the continuous in-depth study of ferroptosis in the development of tumors, especially drug-resistant tumors, people have sought a variety of treatment methods for HNSCC, such as drug therapy, radiation therapy, immunotherapy, nanotherapy, etc. On the basis of these treatments, it is proposed that combination therapy has a good therapeutic effect on HNSCC, especially drug-resistant HNSCC.

However, ferroptosis is a double-edged sword that kills tumor cells while exerting toxicity in other cells. The specific mechanism of ferroptosis in cancer cells and the interaction between ferroptosis and other forms of regulatory death, such as apoptosis, autophagy, and necrosis, remain unclear. Therefore, the precise control of ferroptosis in tumors requires further research. In addition, ferroptosis occurs in normal cells and can eliminate nerve cells (Ayton et al., 2015). Furthermore, ferroptosis can cause tubular epithelial cell death in acute kidney injury (Guo et al., 2021). Ferroptosis can also cause radiation-induced lung injury (Riley, 1994; Basit et al., 2017). Therefore, the use of ferroptosis inducers should focus on treatment timing, dosage, and combination therapy. More advanced pharmacological and immunological studies are needed to evaluate the toxic effects of ferroptosis on normal cells. The pathogenesis mechanism of ferroptosis in HNSCC remains incomplete, and we need to conduct more research focused on HNSCC to find new therapeutic targets.
